# Supplementary material for: A yeast phenomic model for the influence of Warburg metabolism on genetic buffering of doxorubicin
Source: Cancer Metab. 2019 Oct 23;7:9. doi: 10.1186/s40170-019-0201-3 (PMC6806529; doi:10.1186/s40170-019-0201-3)
Supplement: Supplementary file 9 — Additional file 9. GO term-specific heatmaps for REMc/GTF-enriched clusters. GO term-specific heatmaps for significant GO process terms were generated as described in methods and Figs. 3 and 4. Any related child terms are presented in subsequent pages of the parent file name. GO terms with more than 100 children, with 2 or fewer genes annotated to the term, or a file size over 300KB are not shown. All heatmaps are generated with the same layout (see Figs. 3 and 4). [file 40170_2019_201_MOESM9_ESM.bz2 › Additional_File9_GOTermHeatmaps/Additional_File9_GOTermHeatmap/post-Golgi_vesicle-mediated_transport.pdf]

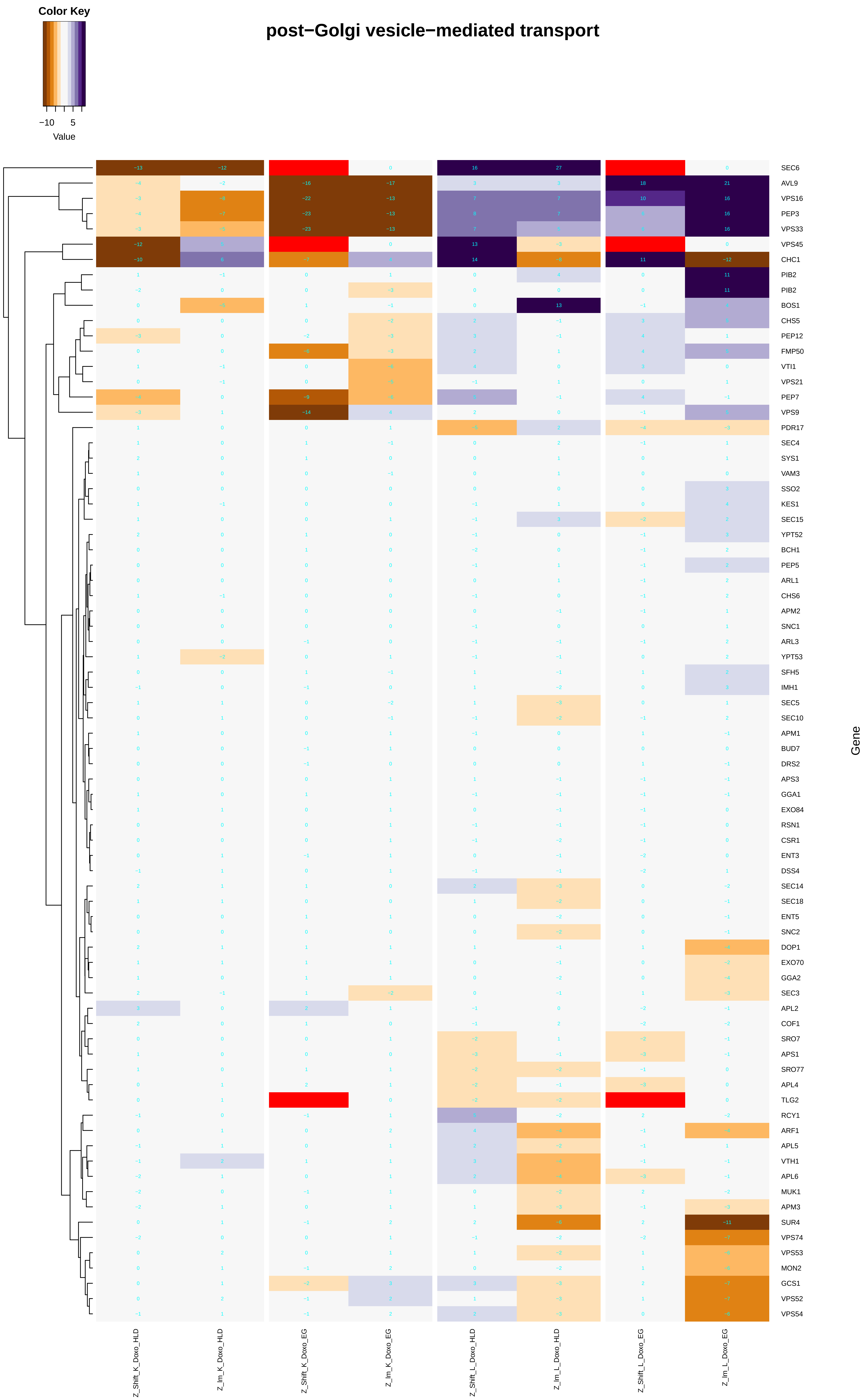

Color Key
−10
5
Value
post−Golgi vesicle−mediated transport
SEC6
AVL9
VPS16
PEP3
VPS33
VPS45
CHC1
PIB2
PIB2
BOS1
CHS5
PEP12
FMP50
VTI1
VPS21
PEP7
VPS9
PDR17
SEC4
SYS1
VAM3
SSO2
KES1
SEC15
YPT52
BCH1
PEP5
ARL1
CHS6
APM2
SNC1
ARL3
YPT53
SFH5
IMH1
SEC5
SEC10
APM1
BUD7
DRS2
APS3
GGA1
EXO84
RSN1
CSR1
ENT3
DSS4
SEC14
SEC18
ENT5
SNC2
DOP1
EXO70
GGA2
SEC3
APL2
COF1
SRO7
APS1
SRO77
APL4
TLG2
RCY1
ARF1
APL5
VTH1
APL6
MUK1
APM3
SUR4
VPS74
VPS53
MON2
GCS1
VPS52
VPS54
Gene
Z_Shift_K_Doxo_HLD
Z_lm_K_Doxo_HLD
Z_Shift_K_Doxo_EG
Z_lm_K_Doxo_EG
Z_Shift_L_Doxo_HLD
Z_lm_L_Doxo_HLD
Z_Shift_L_Doxo_EG
Z_lm_L_Doxo_EG

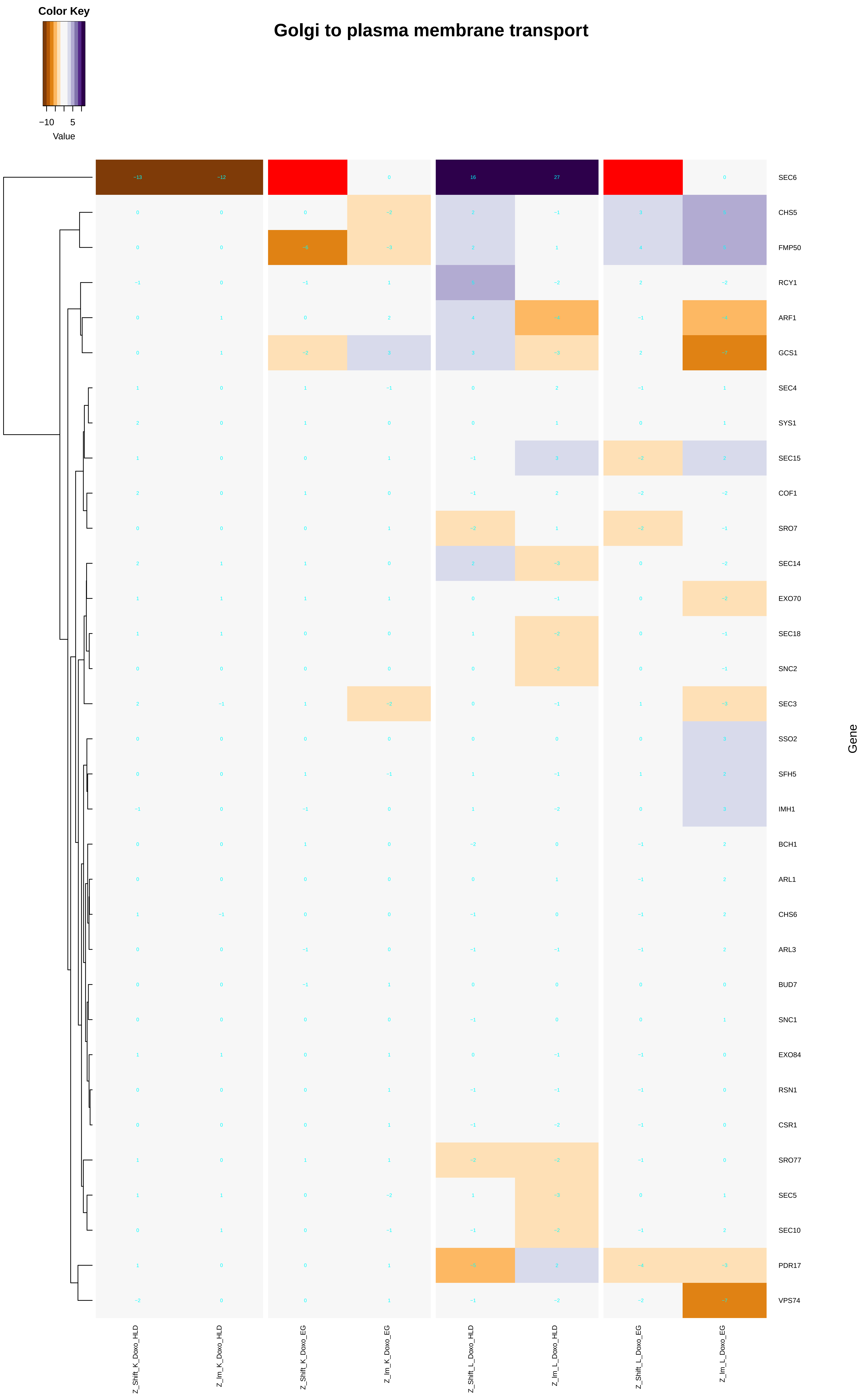
Golgi to plasma membrane transport
Color Key
−10
5
Value
SEC6
CHS5
FMP50
RCY1
ARF1
GCS1
SEC4
SYS1
SEC15
COF1
SRO7
SEC14
EXO70
SEC18
SNC2
SEC3
SSO2
SFH5
IMH1
BCH1
ARL1
CHS6
ARL3
BUD7
SNC1
EXO84
RSN1
CSR1
SRO77
SEC5
SEC10
PDR17
VPS74
Gene
Z_Shift_K_Doxo_HLD
Z_lm_K_Doxo_HLD
Z_Shift_K_Doxo_EG
Z_lm_K_Doxo_EG
Z_Shift_L_Doxo_HLD
Z_lm_L_Doxo_HLD
Z_Shift_L_Doxo_EG
Z_lm_L_Doxo_EG

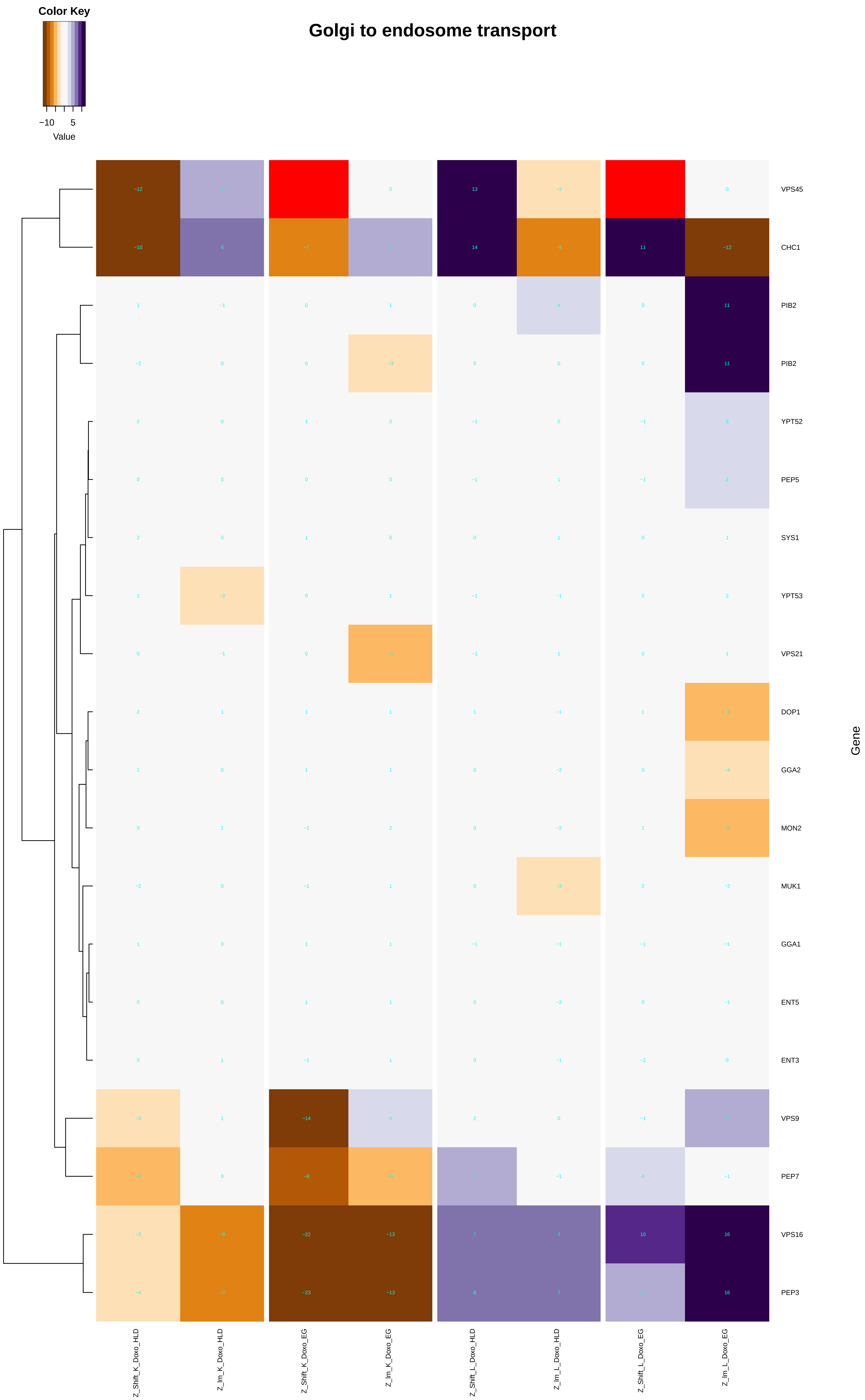

Color Key
−10
5
Value
Golgi to endosome transport
VPS45
CHC1
PIB2
PIB2
YPT52
PEP5
SYS1
YPT53
VPS21
DOP1
GGA2
MON2
MUK1
GGA1
ENT5
ENT3
VPS9
PEP7
VPS16
PEP3
Gene
Z_Shift_K_Doxo_HLD
Z_Im_K_Doxo_HLD
Z_Shift_K_Doxo_EG
Z_Im_K_Doxo_EG
Z_Shift_L_Doxo_HLD
Z_Im_L_Doxo_HLD
Z_Shift_L_Doxo_EG
Z_Im_L_Doxo_EG

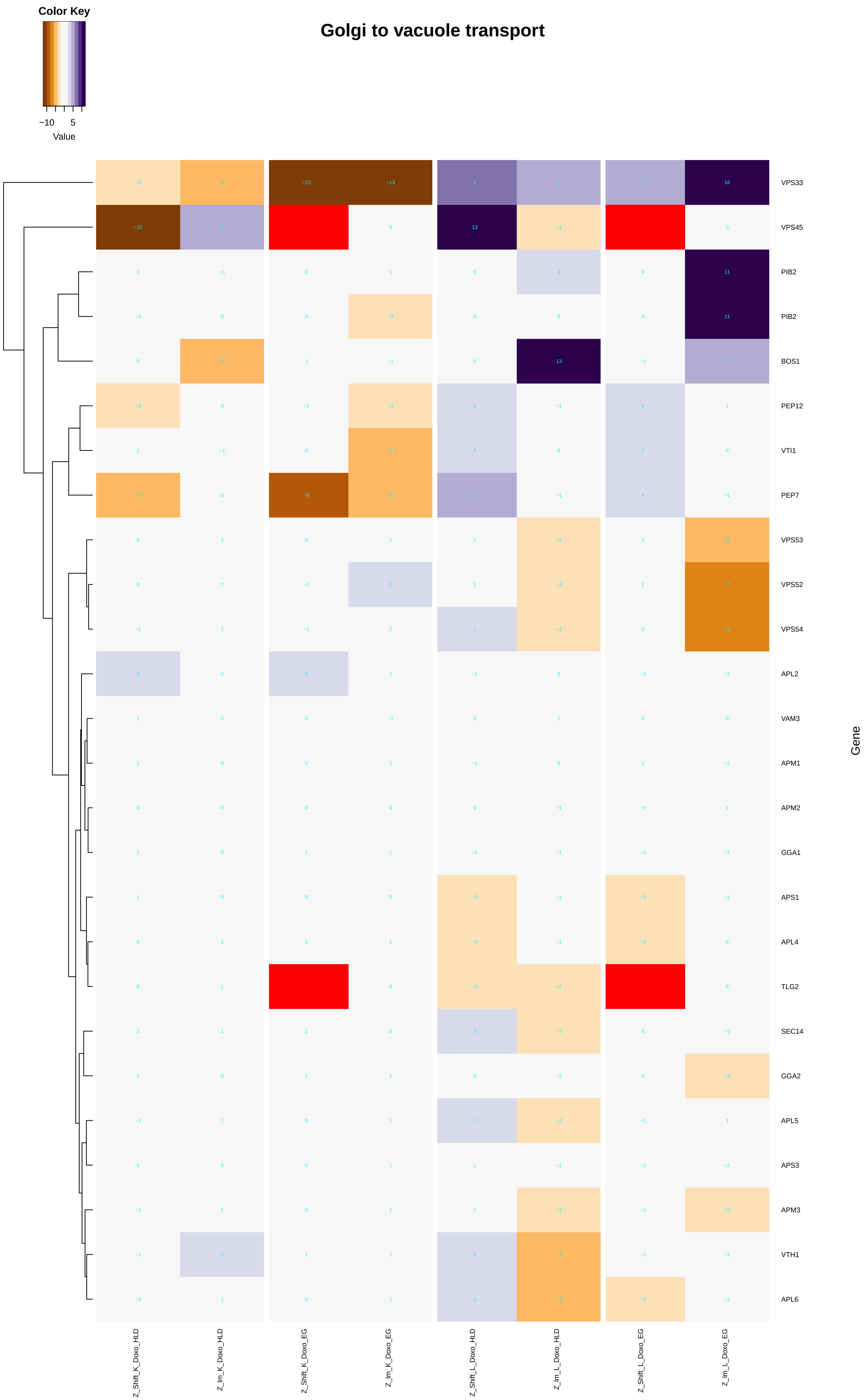
Golgi to vacuole transport
Color Key
−10
5
Value
VPS33
VPS45
PIB2
PIB2
BOS1
PEP12
VTI1
PEP7
VPS53
VPS52
VPS54
APL2
VAM3
APM1
APM2
GGA1
APS1
APL4
TLG2
SEC14
GGA2
APL5
APS3
APM3
VTH1
APL6
Gene
Z_Shift_K_Doxo_HLD
Z_Im_K_Doxo_HLD
Z_Shift_K_Doxo_EG
Z_Im_K_Doxo_EG
Z_Shift_L_Doxo_HLD
Z_Im_L_Doxo_HLD
Z_Shift_L_Doxo_EG
Z_Im_L_Doxo_EG

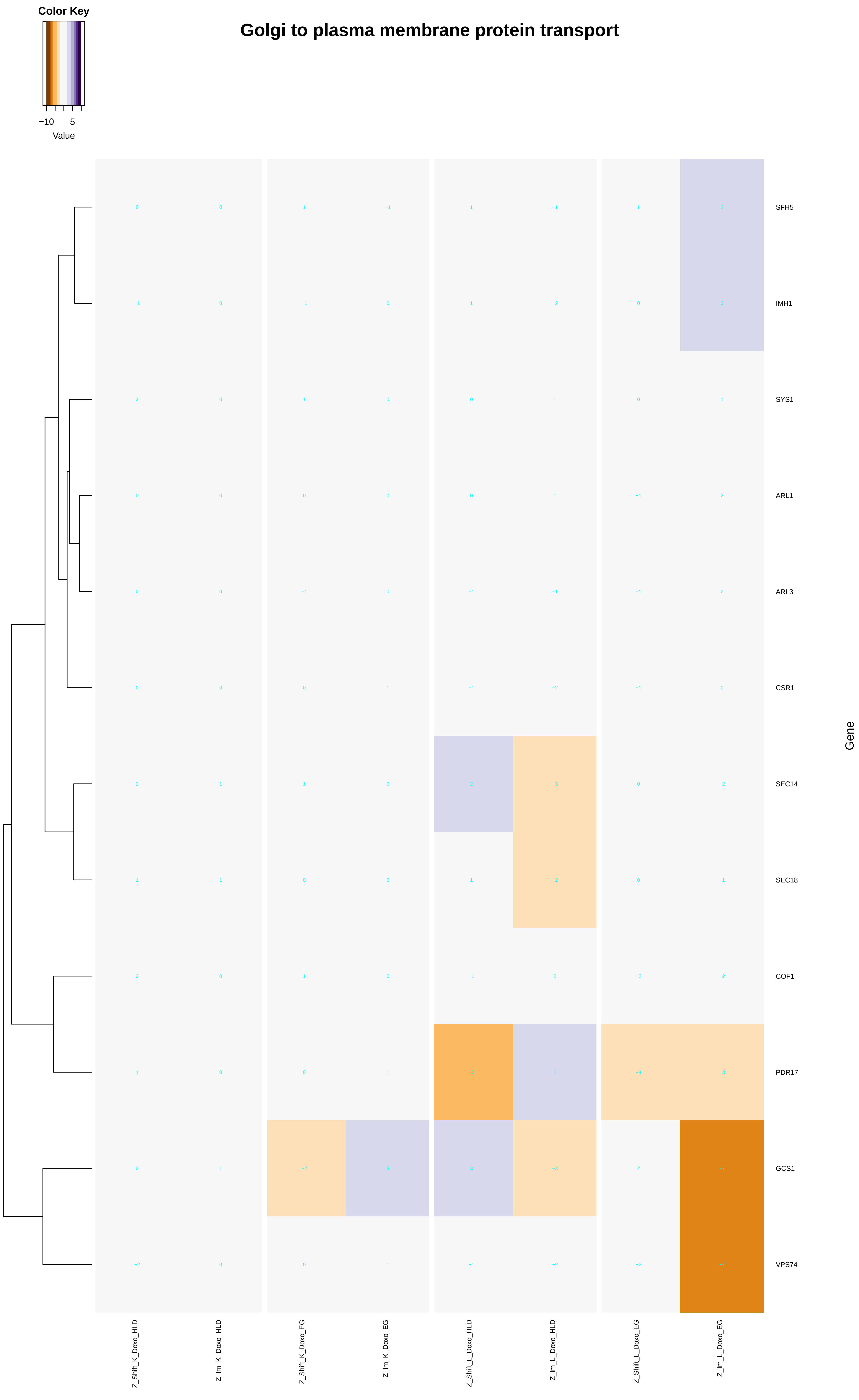
Golgi to plasma membrane protein transport
Color Key
−10
5
Value
Gene
SFH5
IMH1
SYS1
ARL1
ARL3
CSR1
SEC14
SEC18
COF1
PDR17
GCS1
VPS74
Z_Shift_K_Doxo_HLD
Z_Im_K_Doxo_HLD
Z_Shift_K_Doxo_EG
Z_Im_K_Doxo_EG
Z_Shift_L_Doxo_HLD
Z_Im_L_Doxo_HLD
Z_Shift_L_Doxo_EG
Z_Im_L_Doxo_EG
